# Oxidative Stress and Pyroptosis in Doxorubicin-Induced Heart Failure and Atrial Fibrillation

**DOI:** 10.1155/2023/4938287

**Published:** 2023-01-24

**Authors:** Zhang Ping, Tou Fangfang, Zhan Yuliang, Cai Xinyong, Hong Lang, Hu Fan, Ma Jun, Shao Liang

**Affiliations:** ^1^Department of Neurology, Jiangxi Provincial People's Hospital, The First Affiliated Hospital of Nanchang Medical College, Nanchang, 330006 Jiangxi, China; ^2^Department of Cardiology, Jiangxi Provincial People's Hospital, The First Affiliated Hospital of Nanchang Medical College, Nanchang, 330006 Jiangxi, China; ^3^Department of Cardiology, The Second Affiliated Hospital and Yuying Children's Hospital of Wenzhou Medical University, Wenzhou, 325000 Zhejiang, China

## Abstract

Patients undergoing doxorubicin (Dox) chemotherapy often develop new-onset atrial fibrillation and heart failure. Recent studies indicate that the TLR4/MyD88/NLRP3 pyroptosis signaling pathway plays a key role in the occurrence and development of cancer, heart failure, and atherosclerosis. However, few studies investigated the role of oxidative stress and pyroptosis in doxorubicin-induced heart failure and new-onset atrial fibrillation. In this study, we recruited 84 healthy subjects, 112 patients undergoing Dox chemotherapy showing heart failure (HF), and 62 patients undergoing Dox treatment who manifested atrial fibrillation (AF). The mRNA and protein levels of TLR4 expression, several downstream pyroptosis-associated proteins (cleaved caspase-1, NLRP3, GSDMD-N, and HMGB-1), serum inflammatory factors, and oxidative stress were detected at the beginning of chemotherapy and after 3 months of Dox chemotherapy. Oxidative stress and downstream pyroptosis-associated proteins tended to increase in the Dox-baseline group to the Dox-HF group. However, virtually no change in the expression of either oxidative stress or pyroptosis-associated proteins was detected in patients after three months of Dox chemotherapy compared with those at baseline. This study suggests that the prolonged oxidative stress and high levels of pyroptosis-associated proteins contribute to cardiac systolic dysfunction, suggesting TLR4 as a novel biomarker and a potential treatment target for doxorubicin-induced heart failure.

## 1. Introduction

In recent years, early diagnosis of tumors and treatment strategies have prolonged the survival of patients. However, the incidence and mortality due to complications induced by anticancer treatments have increased each year. Cardiovascular disease is the most common adverse reaction of antitumor therapy [1, 2].

Doxorubicin (Dox) is an antitumor drug that is widely used against a variety of malignant tumors, including lymphoma, leukemia, and breast cancer. However, its cardiotoxicity limits its application [3, 4]. The acute cardiotoxicity of doxorubicin can manifest as arrhythmia, while chronic toxicity presents as dilated cardiomyopathy and congestive heart failure. In a recent study, 10.3% of patients undergoing Dox chemotherapy developed new-onset atrial fibrillation. Myocardial damage can be accompanied by rapid cardiac arrhythmia, conduction block, or heart failure. These conditions occur suddenly, without any abnormal signs in the electrocardiogram [3–6].

Pyroptosis is a new type of cell death that depends on the release of proinflammatory factors such as caspase-1, caspase-4, and caspase-5 [7, 8]. Cleaved caspase-1 associates with pattern recognition receptors (toll-like receptors 2 and 4) to form nucleotide-binding oligomerization domain-like receptors or NOD-like receptors (NLRs) and inflammatory bodies during the process of cell death. NLRP3 inflammatory corpuscles enhance inflammation-induced damage in cells by inducing the production of proinflammatory factors and acting as sensors for both invading pathogens and cellular stress during the process of inflammation [9, 10]. Several studies reported that pyroptosis is generally involved in the occurrence and development of cancer, heart failure, radiation injury, chemical damage, and cancer formation and progression [7, 8, 11, 12]. The study of pyroptosis is essential for understanding its role in related diseases and provides new ideas for clinical prevention and treatment.

Toll-like receptors (TLRs) are an important class of endogenous autoimmune factors. To date, more than ten types of TLRs have been found in mammals [13, 14]. TLRs are associated with the development and instability of cardiovascular disease and cancer [15–17]. The TLR protein is a single transmembrane noncatalytic protein that has a conservative molecular structure. TLR4 is a special member of the TLR family, particularly in the context of cardiovascular disease and cancer [18, 19]. A previous study demonstrated that TLR4 knockdown ameliorated Dox-induced heart damage, reduced the oxidant and inflammatory stress response, and reduced cardiac apoptosis [20]. However, it was also found that radiotherapy and chemotherapy caused tumor cell death and the release of high migration rate group protein B1 (HMGB1), which exhibits an effective antitumor immune response mediated via TLR4 receptor and the MyD88 signaling pathway [21].

A balance between oxidant and antiantioxidant mechanisms is obviously important in cancer formation and progression, radiation injury, chemical damage, and other pathological conditions. High expression of reactive oxygen species (ROS) directly exacerbates inflammation, DNA damage, and mitochondrial membrane permeability and even accelerates cellular apoptosis and death [22]. It causes an increase in oxidative stress injury when the cumulative dose of DOX exceeds 500 mg/m^2^. Interestingly, oxidative stress, inflammatory immune response, tumor progression, and heart disease are intricately intertwined and reinforce each other. But there is a lack of researches about oxidative stress and pyroptosis in doxorubicin-induced cardiotoxicity.

In this study, the levels of oxidative stress and pyroptosis as well as the expression of signaling proteins in patients with doxorubicin-induced cardiotoxicity were investigated.

## 2. Materials and Methods

### 2.1. Population Characteristics

All subjects were recruited from the First Affiliated Hospital of Nanchang Medical College and Second Affiliated Hospital of Wenzhou Medical University. The study was approved by the ethics committee of the First Affiliated Hospital of Nanchang Medical College. The study protocol conformed to the principles of the Declaration of Helsinki.

The subjects were divided into the following 2 groups: HF group (84 healthy subjects) and Dox group (174 patients), the latter receiving prior doxorubicin treatment for cancer (lymphomas, leukemia, or multiple myeloma). Three months of doxorubicin therapy included 4 consecutive treatment cycles of cyclophosphamide (750 mg/dL), doxorubicin (60 mg/dL), vincristine (mg/dL), and dexamethasone (15 mg/d 1-5) every 3 weeks. After 3 months of consecutive treatment, 112 patients in the Dox group experienced heart failure without atrial fibrillation (Dox-HF group) and 62 patients in the Dox group were diagnosed with atrial fibrillation without heart failure (Dox-AF group).

The exclusion criteria were as follows: a history of anticancer treatment, heart failure, atrial fibrillation, or other significant arrhythmias and serious neoplastic disease, advanced liver disease, infection, autoimmunity, or pregnancy.

### 2.2. TLR4 mRNA Detection

Patients' blood samples were collected before anticancer chemotherapy and after 3 months of doxorubicin therapy for storage at -80°C. Blood samples were mixed with Ficoll-Paque PLUS reagent and then centrifuged for 20 min at 3000 g.

RNA was extracted from peripheral blood mononuclear cells (PBMCs) with TRIzol and phosphate-buffered saline. The total RNA (more than 400 ng) was reverse-transcribed using a First Strand cDNA Synthesis Kit. The TLR4 mRNA was detected via two-step real-time RT-PCR, and *β*-actin was used as control. The forward and reverse primers of TLR4 were 5′-TGC GGG TTC TAC ATC AAA-3′ and 5′-CCA TCC GAA ATT ATA AGA AA AGT C-3′, respectively. The forward and reverse primer sequences of *β*-actin were 5′-AGC CTC GCC TTT GCC GA-3′ and 5′-CTG GTG CCT GGG GCG-3′, respectively. Thermal cycling conditions were as follows: 95°C for 5 min, 40 cycles of 95°C for 30 s, 56°C for 20 s, 56°C for 20 s, and 72°C for 30 s.

### 2.3. Western Blotting

Blood samples were collected as described before. Proteins in the samples were purified and quantified using the BCA Protein Assay Kit. Precast SDS-PAGE gels were used to separate the sample proteins, and the separated proteins were transferred onto polyvinylidene difluoride membranes. The proteins were blocked with 5% skim milk nonspecifically. The membranes were incubated with the primary antibodies at 4°C the whole night. The primary antibodies included the antihuman TLR4 antibody diluted 1 : 1000 (bs3489, Bioworld Technology, Inc., St. Louis Park, MN, USA), antihuman myeloid differentiation factor 88 1 : 1000 (MyD88, Bioworld Technology, Inc., USA), antihuman NF-*κ*B antibody diluted 1 : 1000 (Abcam, USA), antihuman NLRP3 antibody diluted 1 : 1000 (Abcam, USA), antihuman caspase-1 antibody diluted 1 : 500 (Santa Cruz Inc., USA), antihuman N-terminal fragment of gasdermin D antibody diluted 1 : 500 (GSDMD-N, Abcam, USA), and antihuman HMGB-1 antibody diluted 1 : 500 (Santa Cruz Inc., USA). Corresponding secondary antibodies were used as appropriate prior to exposure.

### 2.4. Serum Inflammatory Factor and Oxidative Stress Markers

Serum was separated from blood samples and frozen at -80°C. Several inflammatory factors, such as MCP-1, IL-1*β*, IL-6, IL-8, TNF-*α*, and TLR4, were detected via respective protocols. TBARS was assayed to reflect the oxidant levels. Antiantioxidant levels of GSH, SOD, GR, and GPx were measured according to the instructions provided by the manufacturers.

### 2.5. Statistical Analysis

All data were expressed as mean ± SD or the median and interquartile ranges for nonparametric data and analyzed with SPSS 13.0 software. One-way ANOVA, followed by Scheffé's test, was performed to analyze continuous data. *P* < 0.05 was considered clinical statistically significant.

## 3. Results

### 3.1. Clinical Characteristics

As shown in Table 1, no significant differences were found in indices such as age, gender, smoking, drinking, hypertension, BMI, and others (*P* > 0.05). However, after three months, the indices of cardiac function were significantly decreased in the Dox-HF group (*P* < 0.05). The history of medicine usage is described in Table 1.

### 3.2. TLR4 and Protein Expression

**Figure 1:**
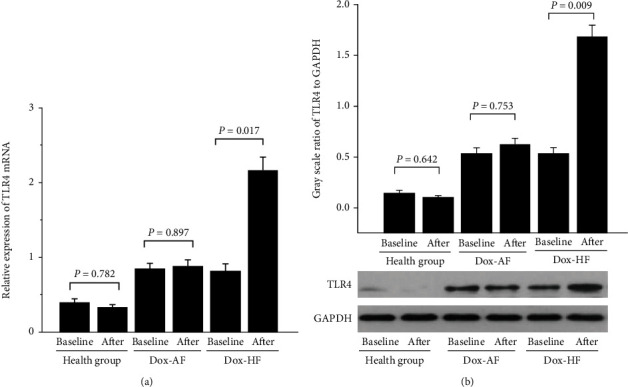
The mRNA and protein expression of TLR4 in Dox patients with AF or with HF. *n* = 84 in health group baseline and after, *n* = 174 in Dox-baseline group, *n* = 62 in Dox-AF after group, and *n* = 112 in Dox-HF after group. *P* < 0.05 indicates a clinical statistically significant difference.

In this study, the TLR4 expression was detected as shown in Figure 1. There was a markedly high expression of TLR4 in the Dox-HF group (mRNA, 2.17 ± 0.11; protein, 1.68 ± 0.18) compared with the Dox-baseline group (mRNA, 0.83 ± 0.04; protein, 0.54 ± 0.06) (mRNA, *P* = 0.017; protein, *P* = 0.009). However, similar expression of both TLR4 mRNA and protein was found in health-baseline and health-after groups, as well as between the Dox-baseline and Dox-AF groups.

**Figure 2:**
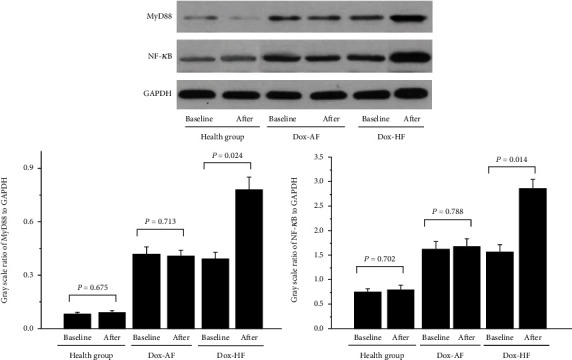
The TLR4 downstream signal protein expression in Dox patients with AF or with HF. *n* = 84 in health group baseline and after, *n* = 174 in Dox-baseline group, *n* = 62 in Dox-AF after group, and *n* = 112 in Dox-HF after group. *P* < 0.05 indicates a clinical statistically significant difference.

Further, after 3 months of doxorubicin therapy, the downstream protein expression of MyD88 and NF-*κ*B was significantly higher in the Dox-HF group (MyD88, 0.78 ± 0.07; NF-*κ*B, 2.87 ± 0.18) than in the Dox-baseline group (MyD88, 0.39 ± 0.04; NF-*κ*B, 1.58 ± 0.14). However, almost no differences were detected in the expression of MyD88 or NF-*κ*B between the health-baseline and health-after groups, as well as between the Dox-baseline and Dox-AF groups, as shown in Figure 2.

### 3.3. Pyroptosis-Associated Protein Expression

**Figure 3:**
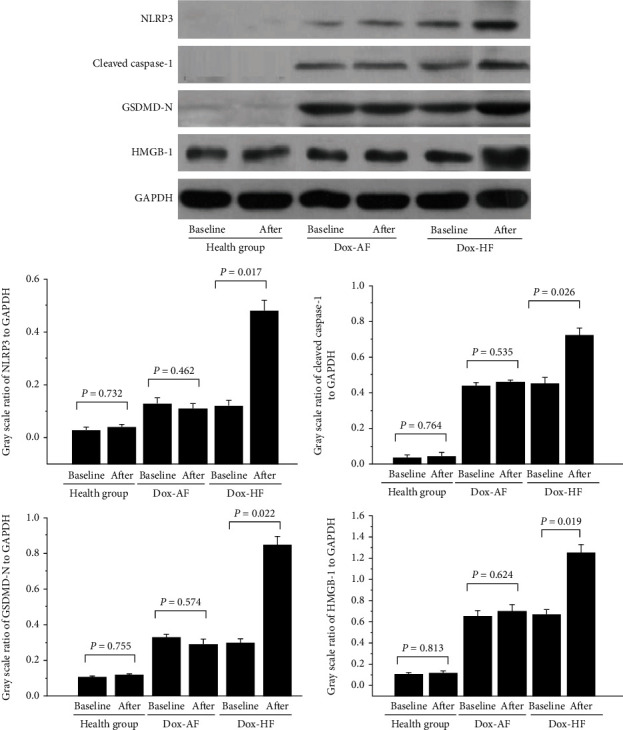
Pyroptosis-associated protein expression in Dox patients with AF or with HF. *n* = 84 in health group baseline and after, *n* = 174 in Dox-baseline group, *n* = 62 in Dox-AF after group, and *n* = 112 in Dox-HF after group. *P* < 0.05 indicates a clinical statistically significant difference.

Pyroptosis plays an important role in Dox-induced cardiotoxicity, as described in Figure 3. In our study, the expression of pyroptosis-associated proteins (cleaved caspase-1, NLRP3, GSDMD-N, and HMGB-1) was detected at baseline and after three months of chemotherapy.

NLRP3 expression was markedly increased after 3 months of doxorubicin therapy in the Dox-HF group (0.48 ± 0.04) compared with the Dox-baseline group (0.13 ± 0.04). There were no differences between the health-baseline (0.03 ± 0.02) and health-after groups (0.04 ± 0.02) or between Dox-baseline (0.14 ± 0.01) and Dox-AF groups (0.11 ± 0.01).

After 3 months of doxorubicin therapy, cleaved caspase-1 expression was markedly increased in the Dox-HF group (0.75 ± 0.08) compared with the Dox-baseline group (0.48 ± 0.04). No differences were found between the health-baseline (0.11 ± 0.03) and health-after groups (0.12 ± 0.01) or between the Dox-baseline (0.45 ± 0.01) and Dox-AF groups (0.47 ± 0.01).

After 3 months of doxorubicin therapy, GSDMD-N expression was markedly increased in the Dox-HF group (0.87 ± 0.21) compared with the Dox-baseline group (0.32 ± 0.09). No differences were found between the health-baseline (0.09 ± 0.01) and health-after groups (0.11 ± 0.03) or between the Dox-baseline (0.33 ± 0.10) and Dox-AF groups (0.31 ± 0.11).

After 3 months of doxorubicin therapy, the NLRP3 expression was markedly increased in the Dox-HF group (1.24 ± 0.14) compared with the NLRP3-baseline group (0.66 ± 0.08). No differences were detected between the health-baseline (0.11 ± 0.04) and health-after groups (0.13 ± 0.01) or between the Dox-baseline (0.67 ± 0.07) and Dox-AF groups (0.69 ± 0.09).

### 3.4. Serum Inflammatory Factors

Further, this study also detected changes in serum inflammatory factors, as shown in Table 2. Most serum inflammation factors (MCP-1, IL-1*β*, IL-6, IL-8, TNF-*α*, and TLR4) were expressed higher in patients after 3 months of doxorubicin therapy with HF compared with the levels in the Dox-baseline groups (MCP-1, *P* = 0.014; IL-1*β*, *P* = 0.017; IL-6, *P* = 0.025; IL-8, *P* = 0.009; TNF-*α*, *P* = 0.021; and TLR4, *P* = 0.024).

However, no differences were found between the health-baseline and health-after groups or between the Dox-baseline and Dox-AF groups.

### 3.5. Serum Oxidant and Antioxidant Levels

Finally, this study also found changes in the oxidant and antioxidant levels, as shown in Table 3, including levels of TBARS, GSH, GR, GPx, and SOD. The expression of serum oxidant biomarkers was higher in patients with HF after 3 months of doxorubicin therapy compared with those in the Dox-baseline groups (*P* = 0.018). However, no difference in TBARS was found between the health-baseline and health-after groups or between the Dox-baseline and Dox-AF groups.

Serum antioxidant biomarkers were lower in patients diagnosed with HF after 3 months of doxorubicin therapy compared with those in the Dox-baseline groups (GSH, *P* = 0.017; GR, *P* = 0.022; GPx, *P* = 0.013; and SOD, *P* = 0.018). However, no differences were found between the health-baseline and health-after groups or between the Dox-baseline and Dox-AF groups.

## 4. Discussion

Recent studies reported oxidative stress- and pyroptosis-induced cell death in both cancer and cardiotoxicity [23, 24]. However, few studies investigated the role of oxidative stress and pyroptosis in Dox-induced heart failure and new-onset AF. Our study indicates that oxidative stress and pyroptosis play an important role in Dox-induced heart failure and new-onset AF. TLR4 may be a potential biomarker of Dox-induced HF.

After three months of Dox chemotherapy, some patients in this study experienced HF without AF, while others were afflicted with AF only. Dox-induced cardiac injury has been attributed to increase oxidative stress and pyroptosis. Recently, we reported that TLR expression is closely correlated with Dox-induced HF [25, 26]. TLR4 also played a key role in pyroptosis linked to HMGB-1, which transmitted signals inducing cell maturation and antigen presentation for activation, as well as activation of NLRP3 inflammasomes to induce inflammation and cell death.

This study reveals interesting findings. The mRNA and protein levels of TLR4 in serum tend to increase from the Dox-baseline group to the Dox-HF group but showed no difference between the health-baseline group and health-after group or between the Dox-baseline and Dox-AF groups. Numerous studies have indicated TLR4 activation during the recruitment of proinflammatory factors, resulting in myocardial damage [18–20]. A similar pattern of TLR4 expression was also found in the downstream expression of MyD88 and NF-*κ*B signaling proteins in patients exposed to Dox chemotherapy. TLR4 binds to the NLRP3 complex to initiate the pyroptotic cascade, thereby further activating the caspase-1-dependent pathway in animal models [27]. Our present study reveals that pyroptosis-associated proteins are increasingly expressed in the Dox-HF group compared with the Dox-baseline group. However, no difference was found between the health-baseline group and the health-after group. Similar to previous animal studies, we found that TLR4 overexpression reactivated downstream MyD88/NF-*κ*B signaling, thereby upregulating the protein expression of caspase-1, NLRP3, HMGB-1, and GSDMD, as well as enhancing serum inflammatory factors in the Dox-HF group, as shown in Table 2.

The expression of TLR4 and pyroptosis varies between doxorubicin-induced HF and new-onset AF. After three months of Dox chemotherapy, almost no change in TLR4 or pyroptosis was detected in patients compared with those at baseline. The mechanism underlying the differences in clinical symptoms after doxorubicin treatment is unknown. Our study suggests that prolonged elevation in the expression of oxidative stress and pyroptosis-related proteins contributes to systolic dysfunction rather than abnormal myocardial electrical activity. Some natural plant extracts or chemically synthesized artificial antioxidants have been tested as DOX-mediated drug therapy for cardiotoxicity. However, there is still a lack of specific biological markers and treatment plan for Dox-induced cardiotoxicity.

Some previous studies have shown an increased mRNA expression of TLR2, TLR3, TLR6, TLR7, and TLR9 in models of acute HF, whereas TLR3, TLR8, and TLR9 levels were increased in chronic myocarditis [28]. TLR4 is the most widely studied and the most promising new target for the treatment of Dox-induced cardiotoxicity via pyroptosis. However, effective prognostic biomarkers and treatment options for Dox-induced cardiotoxicity are unavailable. In conclusion, our study provides a novel biomarker and potential treatment target for doxorubicin-induced HF.

## Figures and Tables

**Table 1:** Clinical patients' characteristics.

	Health group (*n* = 84)	Dox baseline (*n* = 174)	Dox-AF after (*n* = 62)	Dox-HF after (*n* = 112)	*P* value
Age	42 ± 9	42 ± 12	41 ± 11	43 ± 12	0.823
Gender (M/F)	40 : 44	82 : 92	30 : 32	52 : 60	0.914
Smoking	39 (46%)	85 (49%)	31 (50%)	54 (48%)	0.765
Drinking	12 (14%)	31 (18%)	12 (18%)	19 (17%)	0.862
Hypertension	22 (27%)	50 (29%)	19 (31%)	31 (28%)	0.741
BMI (kg/m)	22.37 ± 1.42	22.36 ± 1.45	21.78 ± 1.37	22.15 ± 1.35	0.824
TC (mmol/L)	3.24 ± 0.13	3.64 ± 0.38	3.76 ± 0.41	3.52 ± 0.38	0.713
TG (mmol/L)	1.74 ± 0.23	1.59 ± 0.28	1.56 ± 0.31	1.62 ± 0.19	0.651
HDL cholesterol (*μ*mol/L)	1.27 ± 0.15	1.27 ± 0.22	1.32 ± 0.18	1.22 ± 0.25	0.534
LDL cholesterol (*μ*mol/L)	2.14 ± 0.18	2.46 ± 0.17	1.93 ± 0.21	2.27 ± 0.15	0.658
BUN (mmol/L)	5.18 ± 1.75	5.22 ± 1.63	5.45 ± 1.52	5.07 ± 1.76	0.382
MDRD-eGFR (mL/min/1.73 m^2^)	77.04 ± 5.67	78.56 ± 6.54	78.24 ± 6.07	79.46 ± 7.16	0.627
b-LDH (IU/L)	127.18 ± 20.46	126.09 ± 22.33	124.09 ± 22.33	117.42 ± 21.75	0.674
cTnI (ng/mL)	0.17 (0.05, 0.39)	0.18 (0.08, 0.36)	0.21 (0.10, 0.35)	0.25 (0.07, 0.42)	0.876
NT-proBNP (ng/L)	116.84 ± 12.43	119.46 ± 17.47	125.46 ± 17.31	875.34 ± 52.18	**0.008**
UCG index					
LVEF (%)	65.78 ± 7.54	66.17 ± 5.68	64.81 ± 5.72	40.37 ± 4.24	**0.018**
E/A ratio	1.82 ± 0.18	1.72 ± 0.21	1.69 ± 0.14	0.71 ± 0.13	**0.024**
LVDd (mm)	52.14 ± 3.64	51.78 ± 3.84	51.92 ± 3.97	54.18 ± 4.06	0.623
LVFS (%)	30.17 ± 1.46	29.63 ± 2.15	29.58 ± 2.76	27.79 ± 3.04	0.382
Medicine history					
*β*-Block	6 (7%)	0	47 (76%)	112 (100%)	0
ACEI	8 (9%)	0	12 (19%)	112 (100%)	0
Digoxin	0	0	0	112 (100%)	0
Diuretics	0	0	0	112 (100%)	0

All data were presented as mean ± SD or *n* (%) or median and interquartile range. BMI: body mass index; TC: total cholesterol; TG: triglycerides; HDL: high-density lipoprotein; LDL: low-density lipoprotein; BUN: blood urea nitrogen; MDRD-eGFR: modification of diet in renal disease estimated glomerular filtration rate; b-LDH: blood lactate dehydrogenase; cTnI: cardiac troponin I; NT-proBNP: NT-probrain natriuretic peptide; UCG: ultrasound echocardiogram; LVEF: left ventricular ejection fraction; E/A ratio: ratio of early (E) to late (A) ventricular filling velocities; LVDd: left ventricular end-diastolic diameter; LVFS: left ventricular fractional shortening. *P* value < 0.05 was considered clinical statistically significant.

**Table 2:** Serum inflammation factor.

	TLR4 (pg/mL)	MCP-1 (pg/mL)	TNF-*α* (pg/mL)	IL-1*β* (pg/mL)	IL-6 (pg/mL)	IL-8 (pg/mL)
Health group						
Baseline (*n* = 84)	20.35 ± 1.41	23.56 ± 1.54	12.33 ± 1.09	27.52 ± 1.76	10.74 ± 1.08	24.62 ± 2.15
After (*n* = 84)	21.52 ± 1.37	22.86 ± 1.72	13.42 ± 1.65	28.54 ± 1.91	12.07 ± 1.03	25.38 ± 2.74
Dox-AF group						
Baseline (*n* = 174)	47.46 ± 3.52	57.24 ± 5.63	32.57 ± 4.17	62.75 ± 6.76	30.86 ± 2.18	50.16 ± 5.72
After (*n* = 62)	45.23 ± 4.01	58.32 ± 5.31	32.85 ± 4.38	65.17 ± 7.06	29.63 ± 1.97	49.17 ± 5.83
Dox-HF group						
Baseline (*n* = 174)	48.27 ± 3.77	56.87 ± 5.27	34.34 ± 3.16	64.54 ± 6.81	32.17 ± 2.07	48.33 ± 5.44
After (*n* = 112)	102.34 ± 7.28^∗^	138.92 ± 8.48^∗^	153.46 ± 10.32^∗^	217.42 ± 18.39^∗^	96.57 ± 7.44^∗^	104.35 ± 9.87^∗^

All data were presented as mean ± SD. *P* < 0.05 indicates a clinical statistically significant difference. ^∗^*P* < 0.05 compared with baseline.

**Table 3:** Level of oxidant and antioxidant in serum.

	SOD (U/mg protein)	GSH (*μ*mol/mg protein)	TBARS (nmol/mg protein)	GPx (*μ*mol/mg protein)	GR (*μ*mol/mg protein)
Health group					
Baseline (*n* = 84)	38.36 ± 1.71	35.33 ± 1.64	17.33 ± 0.17	32.44 ± 1.19	35.21 ± 1.41
After (*n* = 84)	37.92 ± 1.38	35.84 ± 1.73	16.82 ± 0.21	32.68 ± 1.27	35.21 ± 1.24
Dox-AF group					
Baseline (*n* = 174)	14.25 ± 2.78	13.72 ± 2.34	35.87 ± 3.12	14.08 ± 3.15	14.25 ± 2.76
After (*n* = 62)	13.17 ± 2.32	15.26 ± 2.73	34.79 ± 3.08	15.47 ± 3.19	16.54 ± 2.67
Dox-HF group					
Baseline (*n* = 174)	14.78 ± 3.26	15.02 ± 3.11	34.02 ± 2.53	16.07 ± 3.44	14.75 ± 3.23
After (*n* = 112)	6.34 ± 1.18^∗^	6.12 ± 1.05^∗^	68.19 ± 6.24^∗^	6.02 ± 1.89^∗^	6.54 ± 1.12^∗^

SOD: superoxide dismutase; GSH: glutathione; TBARS: thiobarbituric acid reactive substances; GPx: glutathione peroxidase; GR: glutathione reductase. All data were presented as mean ± SD. *P* < 0.05 indicates a clinical statistically significant difference. ^∗^*P* < 0.05 compared with baseline.

## Data Availability

The patients' clinical data used to support the findings of this study are restricted by the First Affiliated Hospital of Nanchang Medical College and Second Affiliated Hospital of Wenzhou Medical University in order to protect patient privacy. Data are available from the corresponding author upon request.
